# Hawaiian *Philodoria* (Lepidoptera, Gracillariidae, Ornixolinae) leaf mining moths on *Myrsine* (Primulaceae): two new species and biological data

**DOI:** 10.3897/zookeys.773.21690

**Published:** 2018-07-09

**Authors:** Shigeki Kobayashi, Chris A. Johns, Carlos Lopez-Vaamonde, Camiel Doorenweerd, Atsushi Kawakita, Issei Ohshima, David C. Lees, Sofia Hanabergh, Akito Y. Kawahara

**Affiliations:** 1 Entomological laboratory, Graduate School of life & Environmental Sciences, Osaka Prefecture University, Sakai, Osaka, 599-8531 Japan; 2 McGuire Center for Lepidoptera and Biodiversity, Florida Museum of Natural History, University of Florida, Gainesville, FL 32611 USA; 3 Department of Biology, University of Florida, Gainesville, FL 32611 USA; 4 INRA, UR0633 Zoologie Forestière, F-45075 Orléans, France; 5 Institut de Recherche sur la Biologie de l’Insecte, UMR 7261, CNRS Université de Tours, UFR Sciences et Techniques, Tours, France; 6 Department of Plant and Environmental Protection Sciences, University of Hawaii, 3050 Maile Way, Honolulu, HI 96822 USA; 7 Naturalis Biodiversity Center, PO Box 9517, 2300 RA Leiden, The Netherlands; 8 Center for Ecological Research, Kyoto University, 2-509-3 Hirano, Otsu, Shiga 520-2113 Japan; 9 Department of Life and Environmental Sciences, Kyoto Prefectural University, 1-5 Hangi-cho, Shimogamo, Sakyo-ku, Kyoto 606-8522 Japan; 10 Natural History Museum, Cromwell Road, South Kensington, SW7 5BD, UK; 11 Department of Entomology and Nematology, University of Florida, Gainesville, FL 32611 USA

**Keywords:** DNA barcoding, leaf mine form, *Myrsine knudsenii*, *Myrsine wawraea*, taxonomy

## Abstract

This paper provides new taxonomic and biological data on a complex of gracillariid moths in the endemic genus *Philodoria* Walsingham, 1907 that are associated with *Myrsine* (Primulaceae) in the Hawaiian Islands, United States. Two new species, *Philodoria kauaulaensis* Kobayashi, Johns & Kawahara, **sp. n.** (host: *Myrsine lanaiensis*, *M. lessertiana*, and *M. sandwicensis*) and *P. kolea* Kobayashi, Johns & Kawahara, **sp. n.** (host: *M. lessertiana*) are described. Biological data are provided for two previously described species that also feed on *Myrsine*: *P. auromagnifica* Walsingham, 1907 and *P. succedanea* Walsingham, 1907. For the first time we detail and illustrate genital structures, immature stages, biology, and host plants of *P. auromagnifica* and *P. succedanea*. *Philodoria kolea*, *P. auromagnifica*, and *P. succedanea* occur in sympatry on the island of Hawaii (Big Island), but each species differs in behavioral characters: *P. kolea* utilizes leaves of seedlings and forms a serpentine mine, whereas the latter two utilize leaves of larger plants, and form linear or serpentine to blotch mines. More broadly, leaf mine forms and diagnostic characteristics of the *Myrsine*-feeding species complex of *Philodoria* (as currently known) are reviewed and illustrated.

## Introduction

Hawaii constitutes one of the most geographically isolated archipelagos and harbors thousands of unusual, highly threatened endemic species. Phytophagous insects that rely on endemic Hawaiian plants are of special risk as they depend on the survival of their native host plants. The Hawaiian Islands measure just 0.02% of the area of the United States, but account for nearly 70% of the United States’ historically documented plant and animal extinctions (Wagner et al. 1999). In all, over 360 Hawaiian animal and plant taxa are currently listed as either threatened or endangered under the federal and state Endangered Species Acts. More than 38% of native Hawaiian plants are threatened and 94% of Hawaiian insects are endemic (Evenhuis and Eldredge 1999). Leaf miners have achieved extraordinary localized diversity and are a major component of island ecosystems throughout the Pacific.

*Philodoria* Walsingham, 1907 is a genus of endemic Hawaiian leaf-mining micromoths, containing approximately 30 species, for which the classification remains largely in disarray. The genus can be distinguished from other genera in the Gracillariidae subfamily Ornixolinae by a hindwing with small frenular bristles along the costa in both sexes (Zimmerman 1978, figs 432–435); by a dorsal flap extending from the posterior margin of tergum VIII in the male; and by the female lamella antevaginalis that is sclerotized and semicircular in shape. Many *Philodoria* host plants are threatened along with their native habitat. Indeed, herbarium samples provide one of the few documented cases globally of a probable moth extinction, albeit an undescribed species (Johns et al. 2014). The genus was first described with seven species by Walsingham (1907), and the type species was designated as *P. succedanea* Walsingham, 1907. Zimmerman (1978) published a monograph of Hawaiian insects following Walsingham’s work and many papers by Swezey (1910–1946). Zimmerman divided *Philodoria* into two subgenera, P. (Eophilodoria) and P. (Philodoria), based on the size of the maxillary palpus. His classification was recently rejected by Johns et al. (2016), who constructed a preliminary molecular phylogeny of *Philodoria* based on three genes for 11 *Philodoria* species. In their analyses, the two *Philodoria* subgenera were not monophyletic and morphological characters used to classify them were inferred as homoplasious; the subgenus Eophilodoria Zimmerman, 1978 was established as a subjective junior synonym of the genus *Philodoria* Walsingham, 1907. In addition, Johns et al. (2016) provided new host plant and distribution data for these 11 species. While *Philodoria* was historically treated as similar to *Elachista* (Elachistidae, Gelechioidea), it unequivocally belongs in Gracillariidae (Kawahara et al. 2017) and the genus is unrelated to Gelechioidea (Breinholt et al. 2018). Based on taxon sampling of exemplar gracillariid genera, *Philodoria* appears to be phylogenetically closely related to the ornixoline genus *Chileoptilia* Vargas & Landry, 2005 from Chile (Kawahara et al. 2017).

Larval host plants of *Philodoria* are diverse, with up to six plant orders (Asterales, Apiales, Ericales, Malvales, Myrtales and Rosales) reported as hosts, among which Asterales (Asteraceae: *Dubautia*) and Rosales (Urticaceae: *Pipturus*) appear as dominant hosts (Swezey 1954; Zimmerman 1978). Another host plant that is used by multiple *Philodoria* species is *Myrsine* (Ericales: Primulaceae). According to Zimmerman (1978) and label data from *Philodoria* specimens in the collection of the Bernice Pauahi Bishop Museum (BPBM), there appear to be numerous undescribed *Philodoria* species on *Myrsine*. In total, 19 *Myrsine* species are known to be endemic to the Hawaiian Islands (Wagner et al. 1999), and two species of *Philodoria* that feed on *Myrsine* have been described: *P. succedanea* Walsingham, 1907 (type species of the genus) and *P. auromagnifica* Walsingham, 1907, both with similar scale colors and genital characters (Walsingham 1907, Zimmerman 1978).

In late April 2016, several of the authors collected numerous blotch mines on leaves of *Myrsine* species at two sites on the island of Hawaii (Big Island). Initially, we believed that these mines were created by a single *Philodoria* species, but after studying them, we realized that they comprised diverse larval habits (e.g., forms with spiral or linear mines, larvae in fallen or *in situ* leaves, and some adults which emerged with relatively black forewings). Recent studies (Kawahara et al. 2009, Davis and Wagner 2011, Davis and De Prins 2011, Brito et al. 2013, Moreira et al. 2017) have shown that important diagnostic characters of gracillariids are present in larvae and pupae. However, insufficient early stages have been preserved until now for diagnostics and identification. In this paper, we describe two new species, *Philodoria kauaulaensis* (hosts: *Myrsine lanaiensis*, *M. lessertiana*, and *M. sandwicensis*) and *P. kolea* (host: *M. lessertiana*), and also the genitalic structures, immature stages and new host plant information for the two previously described *Myrsine*-feeding species, *P. succedanea* and *P. auromagnifica*. Four species were reared, and their mine forms and characters are here reviewed and illustrated.

## Materials and methods

### Taxon sampling

All adult moths were reared from leaf mining larvae and their pupal cocoons. Leaf mines and cocoons were collected between 2013–2016 in the locations listed in Table 1. Among the material examined, the final dates refer to the adult emergence and ‘em.’ signifies that an adult emerged and was mounted as a dry pinned specimen; ‘stored’ signifies a dead adult that was stored in 99 % ethanol or RNAlater solution (Thermo Fisher Scientific). Type material designated by Lord Walsingham and specimens collected by Dr K. & Mrs. E. Sattler in the Natural History Museum (NHMUK), and those collected by Mr. O. H. Swezey at the BPBM and the National Museum of Natural History, Smithsonian Institution (USNM) were also examined. Immatures in leaves were reared in plastic cups (420 ml: 129 mm in diameter at top and 60 mm in depth) containing wet cotton at 20 ± 5 °C under a photoperiod condition in the laboratory of 13–16L (hours light) 8–12D (hours darkness).

**Table 1.** Study sites of *Philodoria* species and host plants.

No.	Locality	Island	Collection Longitude and latitude	Elevation (m)	Study Specimens ID	Species name	Host plant
1	Limahuli, Upper Preserve	Kauai	22.1858°N, 158.58°W	900	AYK-HI10-001, 002	Philodoria sp. nr. splendida	Unknown
2	Kokee	Kauai	22.1508°N, 159.6370°W	1230	CJ-433, 442	*P. succedanea*	*Myrsine knudsenii*
3	Kahili	Kauai	No data	400–500	CJ-148	*P. auromagnifica*	*M. wawraea*
4	Mt. Kaala	Oahu	21.4161°N, 158.0997°W	800	CJ-526	*P. succedanea*	*M. lessertiana*
5	Kamakou	Molokai	21.1184°N, 156.9049°W	1170	CJ-241	*P. auromagnifica*	*M. lessertiana*
6	Eke	Maui	20.9379°N, 156.5801°W	870	CJ-136, 531	*P. succedanea*	*M. lessertiana*
7	Kauaula*	Maui	20.8738°N, 156.6183°W	900	CJ-381	*P. kauaulaensis*	*M. lanaiensis*
8	Waikamoi	Maui	20.7826°N, 156.2304°W	1800	CJ-539	*P. succedanea*	*M. lessertiana*
9	Upper Hamakua Ditch Trail	Hawaii	20.0511°N, 155.238°W	900	CLV6239	Philodoria sp. nr. floscula	*Pipturus* sp.
10	Kohala Watershed Partnership	Hawaii	No data	700–1500	CJ-419	*P. succedanea*	*M. sandwicensis*
11	Kaumana Trail	Hawaii	19.45°N, 155.21–155.19°W	900–1000	HILO016	*P. kolea*	*Myrsine* sp.
12	Hawai’i Volcanoes National Park†	Hawaii	9.4138°N, 155.238°W	1090	SKH-5, 10, 13, 15;	*P. succedanea*, *P. auromagnifica*	*M. lessertiana*;
					HILO053, 054, 059;	*P. kolea*;	*Metrosideros polymorpha*
					AYK0001, 0002, CLV6240	*P. basalis*	

Type locality of *P. kauaulaensis and †*P. kolea*

### Morphology and nomenclature

Descriptions focused on the adult stage and leaf mines because of limitations of other material, and because these stages provide a wealth of morphological traits useful for diagnosis. Photographs of leaf mines were taken primarily in the field using Canon EOS 60D and 5D MKIII digital cameras. Some leafmines were scanned using an EPSON Perfection V600 Photo scanner. Observations and measurements were made under a Leica M2 16 dissection microscope at 71–115× and a Leica S6E microscope at 6.3–40× with the aid of a micrometer scale. Images of adults were captured using a Olympus E-330 camera and Moticam 580 5.0 MP. Images were taken at various depths and subsequently stacked using the Helicon Focus 6.22. All images were then edited with Adobe Photoshop Elements 9 into final figures.

For genitalic dissections, the whole abdomen was removed and boiled for 3–4 min in 10% aqueous KOH, and residual scales and soft parts were removed in 70% ethanol. Genitalia were then stained in Chlorazol Black E (1% solution in 70 % ethanol) or acetocarmine for 0.5–1h, dehydrated in a series of 70−100 % ethanol and mounted in Canada balsam on a glass slide.

**Figure 1.**
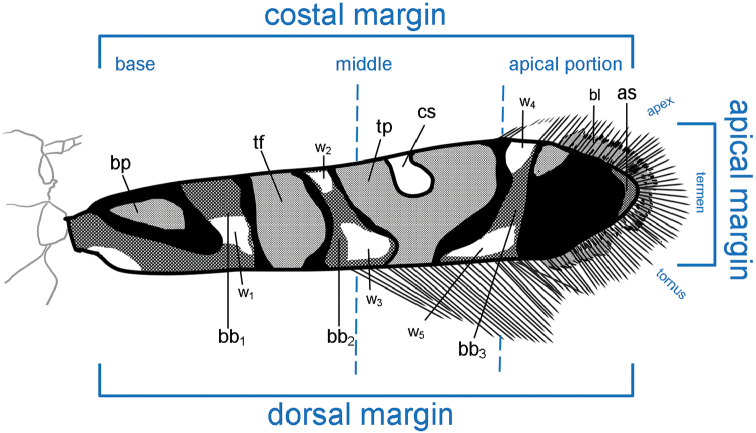
Nomenclature adopted in this study for the characterization of forewing pattern in *Myrsine*-feeding *Philodoria*. Abbreviations: as: apical spot; bb_1_–bb_3_: bronze color band; bp: basal patch; bl: basal line; cs: costal spot; tf: transverse fascia; tp: transverse patch; w_1_– w_5_: white color band.

Type material and additional specimens used in the present study are preserved in the collections of the BPBM, the McGuire Center for Lepidoptera and Biodiversity, Florida Museum of Natural History (FLMNH) and Naturalis Biodiversity Center (RMNH). Terms used for describing wing color pattern are summarized in Fig. 1, and forewing characters follow the terminology of Walsingham (1907) and Zimmerman (1978). Terms for genitalia essentially, follow Zimmerman (1978) and “gnathos” is employed to indicate the sclerotized V-shaped transverse band joining the ventral base of tegumen. Scientific names of plants follow the Plant List (www.theplantlist.org).

### DNA sequencing and analysis

A total of 16 specimens were DNA barcoded. DNA extraction, PCR amplification and sequencing of the 658 base pair Cytochrome Oxidase 1 (COI) “barcode” region for two specimens were carried out at the Canadian Centre for DNA Barcoding (CCDB, Biodiversity Institute of Ontario, University of Guelph) following a published protocol (deWaard et al. 2008). Five specimens were extracted at the Florida Museum of Natural History, McGuire Centre for Lepidoptera and Biodiversity at the University of Florida, Gainesville, FL, USA, using the OmniPrep extraction kit and sequenced at University of Florida’s Interdisciplinary Center for Biotechnology Research (ICBR), one specimen was extracted at the Department of Life and Environmental Sciences, Kyoto Prefectural University, Shimogamo, Kyoto, Japan (KPU) using the DNeasy Blood & Tissue Kit (Qiagen, Inc., Valencia, California), and single-stranded PCR and sequencing for this specimen was carried out at the Operon Sequencing Center following the manufacturer’s protocol (Eurofins, Tokyo, Japan). Eight specimens that were sequenced at Naturalis Biodiversity Center were extracted using a Macherey-Nagel magnetic bead DNA extraction kit on a KingFisher automated DNA extraction robot (Table 2).

**Table 2.** Sampling information of *Philodoria* species used for molecular analysis.

Species name	Collection site	Host plant species	Host plant family	Collection ID	BOLD ID	BOLD BIN	GenBank accession no.	Institution of DNA extraction and sequencing of COI
*P. succedanea*	Hawaii	*Myrsine lessertiana*	Primulaceae	RMNH.INS.30669	WOGRA451-17	ADF5435	MF804823	RMNH, Netherlands
*P. succedanea*	West Maui	*M. lessertiana*	Primulaceae	CJ-144	WOGRA489-17	ADF5435	KT982414	FLMNH, USA
*P. kauaulaensis*	West Maui	*M. lessertiana*	Primulaceae	CJ-064	WOGRA487-17	ADI5327	KT982404	FLMNH, USA
*P. kauaulaensis*	West Maui	*M. sandwicensis*	Primulaceae	CJ-072	WOGRA488-17	ADI5327	KT982407	FLMNH, USA
*P. auromagnifica*	Hawaii	*M. lessertiana*	Primulaceae	RMNH.5013750	WOGRA444-17	ADD6965	MF804828	RMNH, Netherlands
*P. auromagnifica*	Hawaii	*M. lessertiana*	Primulaceae	CLV6240	LEPPC2422-16	ADD6965	MF804824	CCDB, Canada
*P. kolea*	Hawaii	*M. lessertiana*	Primulaceae	IO-322	WOGRA440-17	ADF7137	MF804825	KPU & Eurofins, Japan
*P. kolea*	Hawaii	*M. lessertiana*	Primulaceae	RMNH.INS.30682	WOGRA449-17	ADF7137	MF804831	RMNH, Netherlands
*P. kolea*	Hawaii	*M. lessertiana*	Primulaceae	RMNH.5013751	WOGRA447-17	ADF7137	MF804834	RMNH, Netherlands
*P. kolea*	Hawaii	*M. lessertiana*	Primulaceae	RMNH.5013752	WOGRA448-17	ADF7137	MF804832	RMNH, Netherlands
*P. kolea*	Hawaii	*M. lessertiana*	Primulaceae	RMNH.INS.30684	WOGRA450-17	ADF7137	MF804830	RMNH, Netherlands
Philodoria sp. nr. floscula	Hawaii	*Pipturus* sp.	Urticaceae	CLV6239	LEPPC2421-16	ADD6964	MF804826	CCDB, Canada
Philodoria sp. nr. splendida	Kauai	Unknown	Unknown	AYK-HI10-002	LNOUC1237-11	AAY7555	MF804829	FLMNH, USA
Philodoria sp. nr. splendida	Kauai	Unknown	Unknown	AYK-HI10-001	LNOUC1236-11	AAY7555	MF804827	FLMNH, USA
*P. basalis*	Hawaii	*Metrosideros polymorpha*	Myrtaceae	RMNH.INS.30680	WOGRA446-17	ADF5462	MF804833	RMNH, Netherlands
*P. basalis*	Hawaii	*M. polymorpha*	Myrtaceae	RMNH.5013753	WOGRA445-17	ADF5462	MF804835	RMNH, Netherlands

We conducted an ML analysis of the COI gene using RAxML 8.2.10 (Stamatakis 2014), searching for the best tree using the GTRCAT model and GAMMA-based likelihood optimization for the final tree, and otherwise default settings. Subsequently, 1,000 parametric bootstrap analyses with automated stopping following the extended majority rule criterion were performed to calculate branch support values. Phylogenetic trees were visualized in FigTree 1.4.3 (Rambaut 2009). Intra- and interspecific genetic distances were estimated using the Kimura 2-parameter model implemented within the analytical tools available in BOLDv4 (Table 3). We also used BOLD to obtain Barcode Index Numbers (BINs) (Ratnasingham and Hebert 2013).

**Table 3.** Intra- and interspecific genetic divergences in DNA barcode sequences among studied *Philodoria* species.

Species	*P. succedanea*	*P. kauaulaensis*	*P. auromagnifica*	*P. kolea*	*P. basalis*	Philodoria sp. nr. splendida
*P. succedanea*	[0.88]					
*P. kauaulaensis*	7.0	[0.17]				
*P. auromagnifica*	6.71	5.85	[0.31]			
*P. kolea*	8.91	7.38	8.43	[0.30]		
*P. basalis*	11.12	11.08	10.59	13.28	[1.70]	
Philodoria sp. nr. splendida	13.46	12.10	12.19	13.83	4.41	[1.07]
Philodoria sp. nr. floscula	13.46	15.07	14.78	15.90	13.84	14.93

Kimura 2-parameter (K2P) distances (%) for barcode DNA sequences of the seven analyzed species in the genus *Philodoria*; minimal pairwise distances between species are given for each species pair; values in square brackets represent maximal intraspecific distances.

While single-marker COI analyses can be prone to insufficient resolution and error (Rubinoff and Holland 2005), we were unable to obtain additional genetic data for these species during the time of this study. We therefore chose to use a gene-tree based approach (Hebert et al. 2003; Hajibabaei et al. 2007) as another source of evidence to complement morphology to assess species limits. Sequences, voucher data, images, and trace files are deposited in the Barcode of Life Data Systems (BOLD) (Ratnasingham and Hebert 2007; www.barcodinglife.org). Furthermore, all sequences are deposited in GenBank, and are available as a single dataset DS-PHDRIA (http://dx.doi.org/10.5883/DS-PHDRIA)

### Voucher specimen numbers

Institutional voucher numbers are given here for primary type material and museum collections. In the cases of NHMUK/BMNH numbers, for clarity and consitency they are cited without a space nor hash symbol (#) that might be read between the alpha and numeric parts of the code, since spaces and hashes create ambiguity for search, and series of institutional numbers have appeared in the past with or without such symbols

Abbreviations for collections:

**BPBM** Bernice P. Bishop Museum, Department of Zoology, 1355 Kalihi Street, Honolulu, Hawaii 96818, USA.

**FLMNH** McGuire Center for Lepidoptera and Biodiversity, Florida Museum of Natural History, University of Florida, Gainesville, FL 32611, USA.

**NHMUK** Natural History Museum, Department of Zoology, Cromwell Road, London SW7 5BD, United Kingdom (formerly the British Museum [Natural History] or BMNH).

**RMNH** Naturalis Biodiversity Center, PO Box 9517, 2300 RA Leiden, Netherlands.

**USNM** National Museum of Natural History, Smithsonian Institution, 10^th^ St. & Constitution Ave. NW, Washington, DC 20560, USA.

## Results

### Key to adults

**Table:** 

1	Forewing leaden grey, externally with fuscous brown (Fig. 3)	***P. kolea* sp. n.**
–	Forewing shiny, metallic bronze with bright to dark orange patches	**2**
2	A bright orange transverse fascia at 3/4 in middle interrupted with blue patch; an orange medial transverse fascia, narrowing towards dorsum, (Figs 5I, J, 10E, F)	***P. kauaulaensis* sp. n.**
–	An orange transverse patch beyond middle to costal 3/4, narrowing towards dorsum, extending to dorsal 2/3, with white costal spot	**3**
3	A black patch along costal fold (Figs 5C, D); a fuscous patch near apex (Figs 1A, B, 8A)	***P. succedanea***
–	An orange patch along costal fold, fringed with blackish scales (Figs 1E, F, 5F, H); a fuscous patch with dark orange scales at apex	***P. auromagnifica***

### Key to male genitalia*

**Table:** 

1	Saccus slender, curved toward dorsum (Fig. 6B); vinculum small and inflexed on the ventral side (Fig. 6C)	***P. succedanea***
–	Saccus broad and straight (Fig. 6F, J); vinculum large and inflexed on the ventral side (Fig. 6G, K)	**2**
2	Valva slightly narrowing in middle with terminally rounded dorsal process (Fig. 6E)	***P. auromagnifica***
–	Valva with short, pointed dorsal process (Fig. 7A)	***P. kolea* sp. n.**
	*Male of *kauaulaensis* is unknown.

### Key to female genitalia

**Table:** 

1	Signa with minute spines (Fig. 7I)	***P. kolea* sp. n.**
–	Signa with a pair of larger spines	**2**
2	Spines long and slender (Fig. 7E, F)	***P. succedanea***
–	Spines on the signa small and rounded (Fig. 7H)	***P. kauaulaensis* sp. n.**
–	Spines on the signa blunt (Fig. 7G)	***P. auromagnifica***

### Key to leaf mines

**Table:** 

1	Start of mine spiral-shape (Fig. 10B, C). Mines on *Myrsine lanaiensis*, *M. lessertiana*, *M. sandwicensis*; Maui	***P. kauaulaensis* sp. n.**
–	Start of mine linear or serpentine-shape	**2**
2	Reddish brown long linear mine following leaf vein (Fig. 9D–F), mature larvae in fallen leaves (Fig. 9A). Mines on *M. knudsenii*, *M. lessertiana*, *M. linearifolia, M. sandwicensis*; Kauai, Oahu, Lanai, Maui, Hawaii	***P. succedanea***
–	Brown serpentine mines, mature larvae in situ leaves	**3**
3	Larvae utilize leaves on larger plants. Mines on *M. lessertiana*, *M. sandwicensis*, *M. wawraea*; Kauai, Oahu, Molokai, Hawaii	***P. auromagnifica***
–	Larvae utilize leaves on seedlings (Figs 12A, E, 13D). Mines on *M. lessertiana*; Hawaii (Big Island)	***P. kolea* sp. n.**


#### Philodoria succedanea

Taxon classificationAnimaliaLepidopteraGracillariidae

Walsingham, 1907

**Figure 2.**
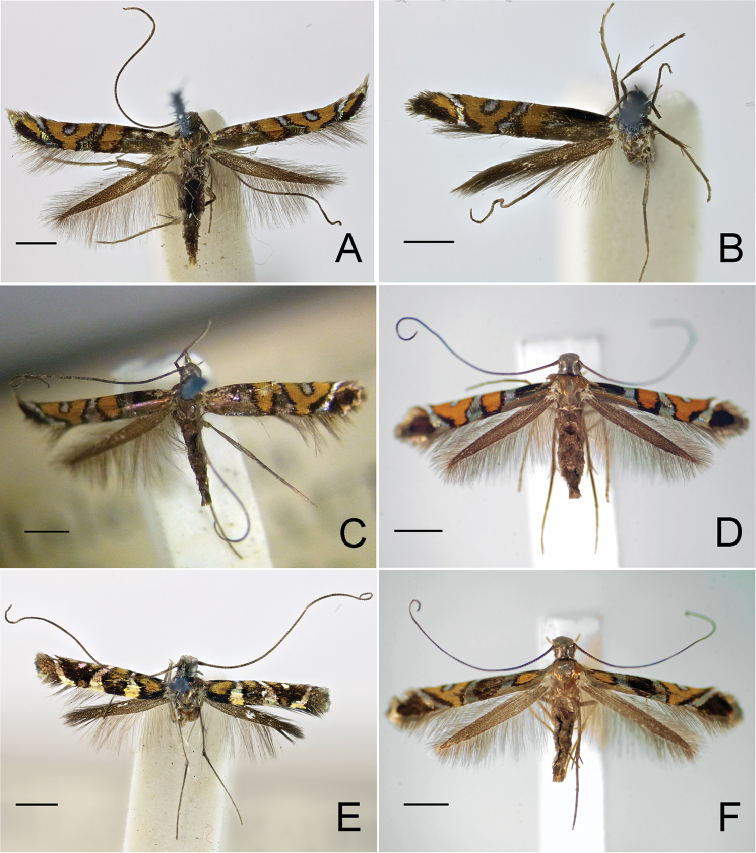
Adults of *Myrsine*-feeding *Philodoria* species. **A–D** *P. succedanea* Walsingham, 1907 **A** Lectotype female **B** Paralectotype male **C** Paralectotype female **D** Male Lanai **E–F** *P. auromagnifica* Walsingham, 1907 **E** Holotype male **F** Female Kauai. Scale bar: 1 mm.

Figs 2A–D , 5A–D , 6A–D , 7E, F , 8A , 9 , 14A

Philodoria succedanea Walsingham, 1907: 717–718; pl. 25, fig. 19.
Philodoria (Philodoria) succedanea Walsingham, 1907: Zimmerman 1978: 718, figs 433, 435, 467, 472.

##### Type locality.

Olinda, Haleakala (Maui).

##### Type material.

**Lectotype** ♀, Olinda, 4000 ft., Haleakala, MAUI, Hawaiian Is. iv.1894, Perkins. 26695 [Walsingham specimen number]|PHILODORIA SUCCEDANEA Wlsm. Fn. Hawaii. I TYPE ♀ descr. fig^d^.|Walsingham Collection. 1910-427.|NHMUK010305341 (here designated).

**Paralectotypes** 17 (2♂ 1♀ 14 unsexed; NHMUK ones are all from above Walsingham accession and ‘PARATYPE’ below is short for ‘PHILODORIA SUCCEDANEA Wlsm. PARATYPE’ as printed on large black-margined labels, with the 5-digit Walsingham specimen numbers whose first digit is ‘2’ borne on the locality label): 1 ♂, Haleakula 4000 ft. MAUI, Hawaiian Is. V. 1896|Perkins. 28505|PHILODORIA SUCCEDANEA Wlsm. Fn. Hawaii. I TYPE ♂|BM ♂ Genitalia slide no. 2755|NHMUK010305341. 1♀ 2 unsexed: Haleakala, 5000ft, MAUI, Hawaiian Is., v.1896, Perkins. 28355|PARATYPE 3/17|NHMUK010862804|; 28230|PARATYPE 4/17♀|BPBM 34324; 28236|PARATYPE 5/17|BPBM 34321. 4 unsexed: Haleakula -4000 ft. Maui, v. 1896, Perkins. 28492|PARATYPE8/17; 28493|PARATYPE 9/17|BPBM 34320|; 28494|PARATYPE10/17|NHMUK010862806; 28495|PARATYPE 11/17|NHMUK010862807.

1 ♂ 7 unsexed, same data and locality as lectotype: 26696|NHMUK010862803; 26661|PARATYPE1/17|BPBM34325; 26667|PARATYPE2/17|BPBM 34222; 28511|PARATYPE12/17|NHMUK010862808; 28512|PARATYPE13/17|NHMUK010862809; 28513|PARATYPE14/17|NHMUK010862810; 28514|PARATYPE15/17♂|NHMUK010862811; 28552|PARATYPE 16/17|BPBM 34323.

This species was described from 19 specimens: ‘type ♀ (26695); ♂ (28505)’ and 17 ‘paratypes’ from Kauai and Haleakala, Maui. This seems to indicate that Lord Walsingham considered them as holotype, allotype, and paratypes, as indicated on their specimen labels. But as a holotype was not specified in the description, the so-labelled types and paratypes are all to be considered syntypes under the present Code, Article 73.2 (ICZN 1999), and any one is thus eligible for designation as lectotype. The syntype ‘type ♀ (26695)’, which Walsingham listed first and figured, is here designated as lectotype (Fig. 2A). The remaining syntypes are therefore paralectotypes.

##### Additional material.

32 (11♂ 15♀ 6 unsexed).

Adults: Oahu Is.: 2♀, Mt. Kaala, 18.ii.1923, Swezey coll., host: “*Suttonia*” (= *Myrsine*), SK797♀, 798♀ in USNM; 3♂ 3♀, Palikea, 28.iii.2016 stored in 99 % ethanol (stored), K. Bustamente leg., host: *M. lessertiana*, 10.xi.2015, CJ-526, CJ-531, SK639♂, SK803♀, 804♀, 807♂, 808♂ in BPBM.

Molokai Is.: 1♀, Kainalu [Kainalu Forest, South East Molokai Forest Reserve], 27?vii.1927, *Philodoria auromagnifica* Walsingham Det. by O.H. Swezey, 34145 in BPBM; 1 unsexed, 4000 ft Molokai P. 2.02 *Philodoria succedanea* Wals. 1/1 E. Meyrick det. in Meyrick coll., in NHMUK.

Lanai Is.: 2♂, 2750 ft, Munro Trail, 2.x.1976, K. & E. Sattler BM1976-605, BMNH(E)1621676 and BMNH(E)1621677, *Philodoria* sp. 8 (Lanai) Sattler coll. D.C. Lees Sep 2016.

Maui Is., in BPBM: 1♂ 6♀ 1 unsexed, below Eke, 17&21.v.2013 (stored), C.A. Johns leg., host: *Myrsine* sp., 24.iv.2013, CJ-136, 141, SK799♀; 1♂, Waikamoi, 24.v.2016 (stored), C.A. Johns leg., Spring.2016, CJ-539, SK641♂.

Hawaii Is., host: *M. lessertiana* in BPBM: 1♂ 1♀, Hawai’i Volcanoes National Park, Hawaii, A. Kawakita leg., “Leaf-dropper”, 25.iv.2016 (larva), SK624♀, SK625♂; 2♂ 1♀ 1 unsexed, Same locality, 17&21.v.2016 em., A.Y. Kawahara leg., 29.iv.2016(Cocoon & larva), SKH-10, SK801♂, SKH-13, SK633♂. 1♀, 3800 ft, N. Kohala, Distr. Kohala Mts, Puu Laalaau area, 14–17.vii.1976, K. & E. Sattler, BM1976-605, BMNH(E)1621089, *Philodoria* sp. 9 (Hawaii) Sattler coll. D.C. Lees Sep 2016, 1621676 in NHMUK; 1♂, same data as last specimen, BMNH(E)1621090, *Philodoria* sp. 9 (Hawaii) Sattler coll. D.C. Lees Sep 2016; 1 unsexed, Kohala Watershed Partnership, 9.vi.2015 (stored), C.A. Johns leg., host: *Myrsine sandwicensis*, 18.v.2015, CJ-419 in BPBM.

Larvae: 2 unsexed, Kokee, Kauai Is., 16&26.vi.2015 (stored), C.A. Johns leg., host: *M. knudsenii* 15.vi.2015 (larva), CJ-433, 442 in FLMNH.

##### Diagnosis.

This species is very similar to *P. auromagnifica* feeding on the same hostplant, *Myrsine*, but is recognizable by the rather bright orange patches and black triangular shaped basal patch in the forewing (Table 4; Figs 2A–D, 5A–D); in the male genitalia by the rather broad valva, slender and long saccus curving toward dorsal side (Fig. 6A–C); in the female genitalia by signa with slender and long spines (Fig. 7E, F).

**Table 4.** Diagnostic features of four *Myrsine*-mining *Philodoria* species.

Species name	*P. succedanea*	*P. kauaulaensis*	*P. auromagnifica*	*P. kolea*
Forewing	Shiny, metallic bronze with bright orange-ochreous	Similar to *P. succedanea*	Shiny, metallic bronze with dark brownish orange	Leaden grey, externally with brownish fuscous
Basal patch	Black, triangular-shape	Absent, orange transverse fascia from costal fold to dorsal 1/4	Brownish orange with black ground color, sometimes black	Brownish fuscous
Apical orange transverse fascia	Absent	Present	Absent	Absent
Apical portion	Fuscous, sometimes orangish encroaches on the apex	Fuscous	Fusocus with dark orange scales	Leaden gray
Genitalia				
Valva	Broad	Unknown	Rather long and narrowing in the middle	having rather shorter and pointed dorsal process
Vinculum	Small, inflexed on the ventral side	Unknown	Large, inflexed on the ventral side	Small, inflexed on the ventral side
Saccus	Slender and long, curved toward dorsal side	Unknown	Broad and straight	Broad and straight
Spine on signum	Long and slender	Rather smaller and rounded	Rather blunt	Minute
Distribution^a,b^	**Kauai**, **Oahu**, **Lanai**, Maui, Hawaii	Maui	**Kauai**, Oahu, Molokai, Hawaii	Hawaii
Host plant species^a,b^	*Myrsine lessertiana*, *M. sandwicensis*, ***M. knudsenii***, ***M. linearifolia***, *Myrsine* sp.	*Myrsine lessertiana*, *M. lanaiensis*, *M. sandwicensis*	*Myrsine lessertiana*, *M. sandwicensis*, ***M. wawraea***, *Myrsine* sp.	*Myrsine lessertiana*
Larval habit type	Leaf dropper	Unknown (probably non leaf dropper)	Non leaf dropper	Non leaf dropper
Mining form	Long, linear, along leaf vein	At first spiral, later blotch	Serpentine	Serpentine
Mine color	Red	Brown	Brown	Brown

^a^ As indicated by published data (Zimmerman 1978 and Johns et al. 2016) and see s also pecies descripution.^b^ Plant species and island name in bold indicate new records in the present study. Islands underlined denote type-locality islands.

##### Redescription.

**Adult** (Figs 2A–D, 5A–D, 8A). Wingspan 9–10 mm in type series; forewing length 4 mm in 'TYPE ♂ (28505)' (fig. 2B), 3.6–3.8 mm in paralectotypes. Head bronze; frons white; maxillary palpus reduced; labial palpus bronze grey, with dark brown scales at apex. Antenna shiny tawny fuscous. Thorax bronze.

Forewing shiny, metallic bronze with bright orange-ochreous patches: a black triangular basal patch along the costal fold (Figs 5A, C, 8A); an oblique transverse fascia before the middle of wing, bordered with black scales; a large transverse patch after the middle to costal 3/4, distinctly narrowing in the dorsum, extending to dorsal 2/3, containing white costal spot; one white color band on the middle of the first bronze color band, others on both extremities of second and third bands; a fuscous patch extending toward the termen and apex with a black apical spot, sometimes with orange-ochreous color encroaching on the apical part; cilia tawny, with two metallic silver basal lines, one at the apical cilia, another from termen to tornus. Hindwing dark tawny; cilia tawny. Abdomen tawny above, silvery beneath. Legs tawny, with silvery spurs and slightly paler tarsi.

**Male genitalia** (Fig. 6A–D) (n = 7). Capsule 960 µm. Uncus absent. Tegumen 570–580 µm long, 1.2–1.3× length of valva with series of long hairs at lateral side of base (Fig. 6C). Tuba analis membranous with weakly sclerotized subscaphium; gnathos V-shaped transverse band, terminal margin weakly joining subscaphium and anterior process connecting ventral base of tegumen. Valva broad, 430 µm in length covered with fine setae distally, and having a short dorsal process (Fig. 6A).Vinculum U-shaped; saccus 250 µm long, slender , curved toward dorsal side (Fig. 6B, C). Phallus 720 µm long, tubular and long about 1.2–1.3× length of valva, sinuous in lateral view with two series of minute spiniform cornuti in vesica; coecum slightly curved toward inner side (Fig. 6D).

**Female genitalia** (Fig. 7E, F) (n = 7). Ostium bursae rather small, opening at the middle of 7^th^ abdominal segment; antrum cup-shaped with slender a pair of lateral lobes; ductus bursae slender, tubular, extremity connected to antrum very slender and membranous, curved inside of body, and middle part weakly sclerotized and plate-shape; end of the ductus bursae broad; inception of ductus seminalis on the posterior part of ductus bursae. Corpus bursae pyriform, anterior end weakly sclerotized; some lines consisting of wrinkles running longitudinally, some sclerotized; paired signa with a pair of long slender spines.

**Figure 3.**
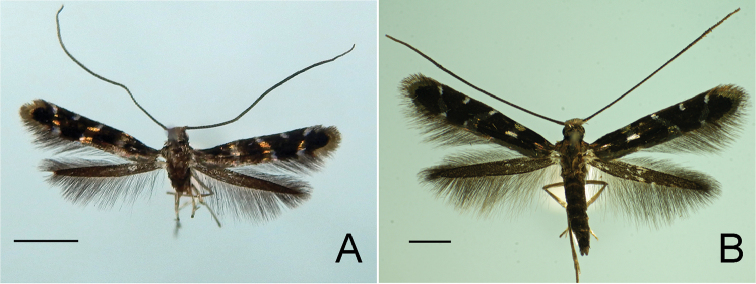
Adults of *P. kolea* sp. n. **A** holotype male **B** Paratype female. Scale bar 1 mm.

**Figure 4.**
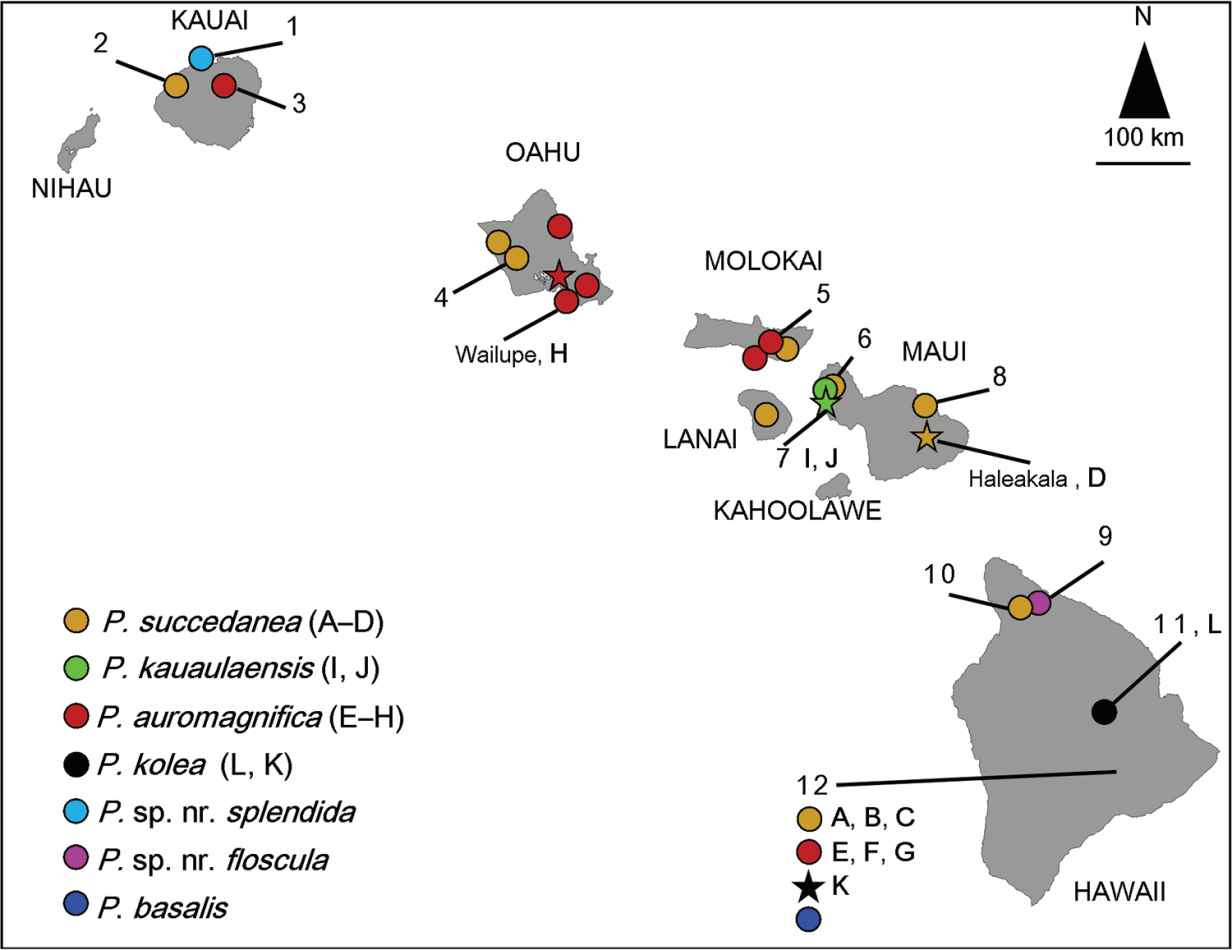
Distribution of *Myrsine*-feeding *Philodoria* species. The star indicates the type locality of each species. Information based on this study and label data of specimens in BPBM, USNM, and NHMUK. Symbols are numbered according to showing locality in Table 1 and alphabetical symbols (A–K) correspond to figure numbers in Figure 5.

**Figure 5.**
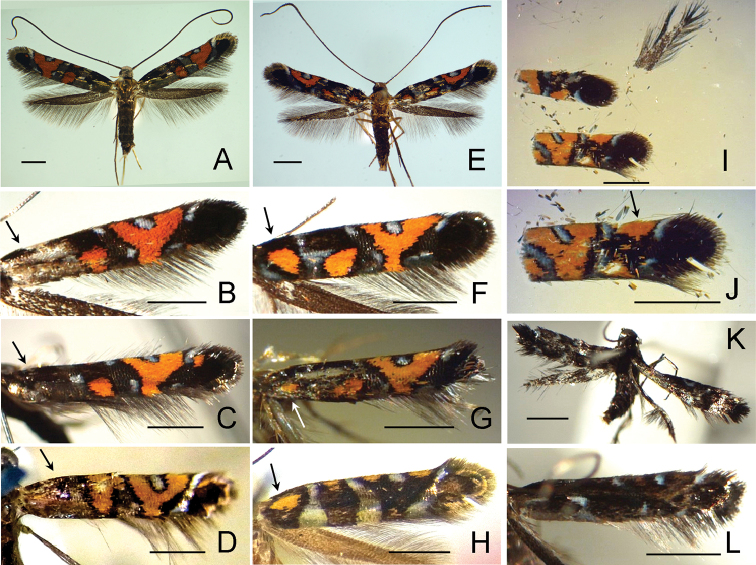
Forewing coloration and pattern of *Myrsine*-feeding *Philodoria* species. **A–D** *P. succedanea* **E–H** *P. auromagnifica* **I, J** *P. kauaulaensis* **K, L** *P. kolea*. **A–C, E–G, K** Hawai’i Volcanoes National Park. **A** Female SK622, leaf-dropper **B** Male SK625, leaf-dropper **C** Male SK633 **D** Paralectotype female 34320 Haleakala, Maui in BPBM **E** Female SK624, non-leaf-dropper **F** Female SK623, non-leaf-dropper **G** Male SK805 **H** Female Z-XII-20-62-6 34143 Wailupe, Oahu, in BPBM **I, J** Holotype female SK690 **K** Paratype female SK632 **L** Paratype female SK631. Scale bars: 1 mm.

**Figure 6.**
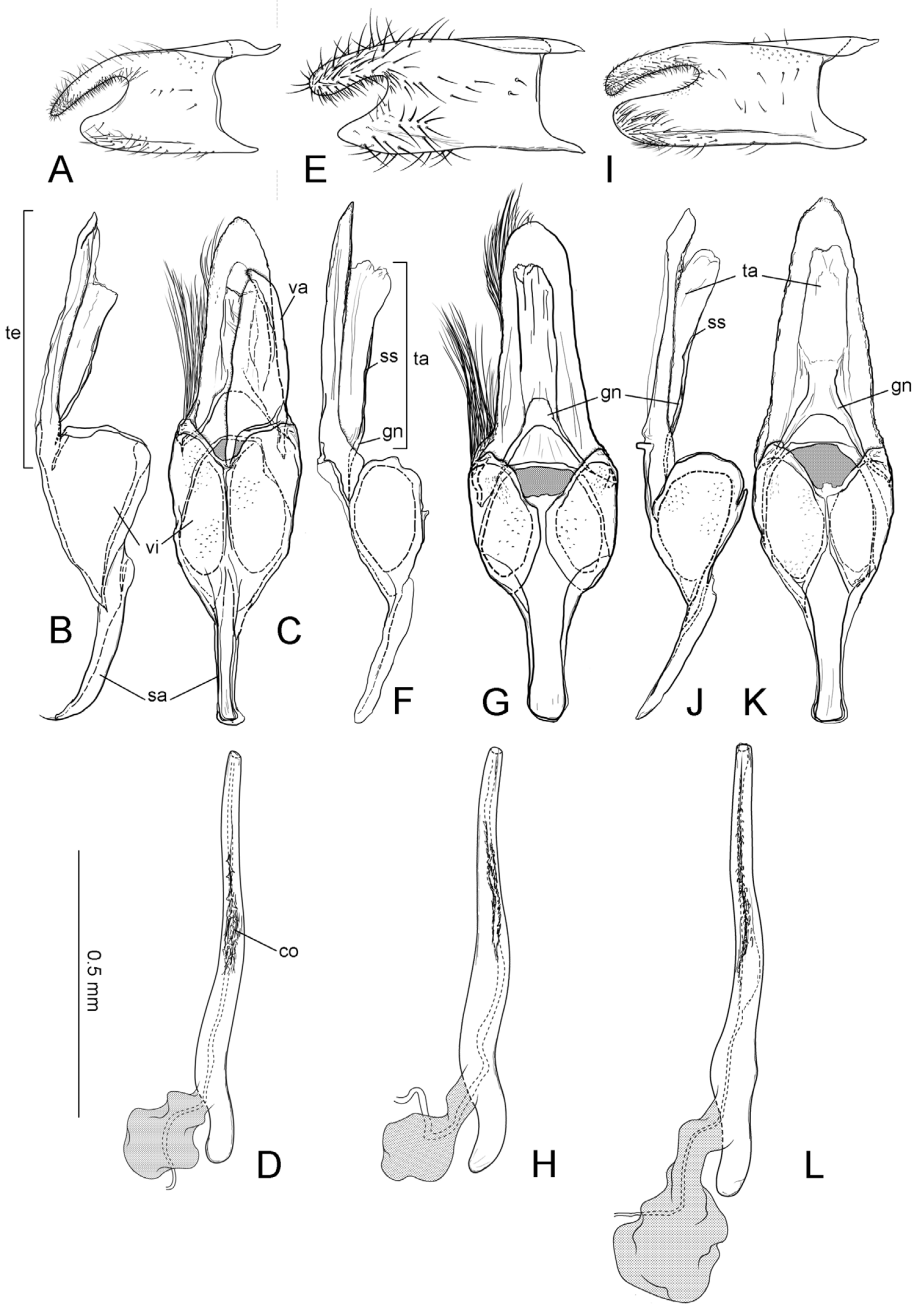
Male genitalia of *Philodoria*. **A–D** *P. succedanea* Maui SK641 **E–H** *P. auromagnifica* Hawaii SK800 **I–L** *P. auromagnifica* Kauai SK689 **A, E, I** Left valva **B, F, J** Genital capsule lateral view **C** Genital capsule with left valva ventral view **G, K** Genital capsule ventral view **D, H, L** Phallus lateral view. Abbreviations: co: cornuti; gn: gnathos; sa: saccus; ss: subscaphium; ta: tuba analis; te: tegumen; va: valva; vi: vinculum.

**Figure 7.**
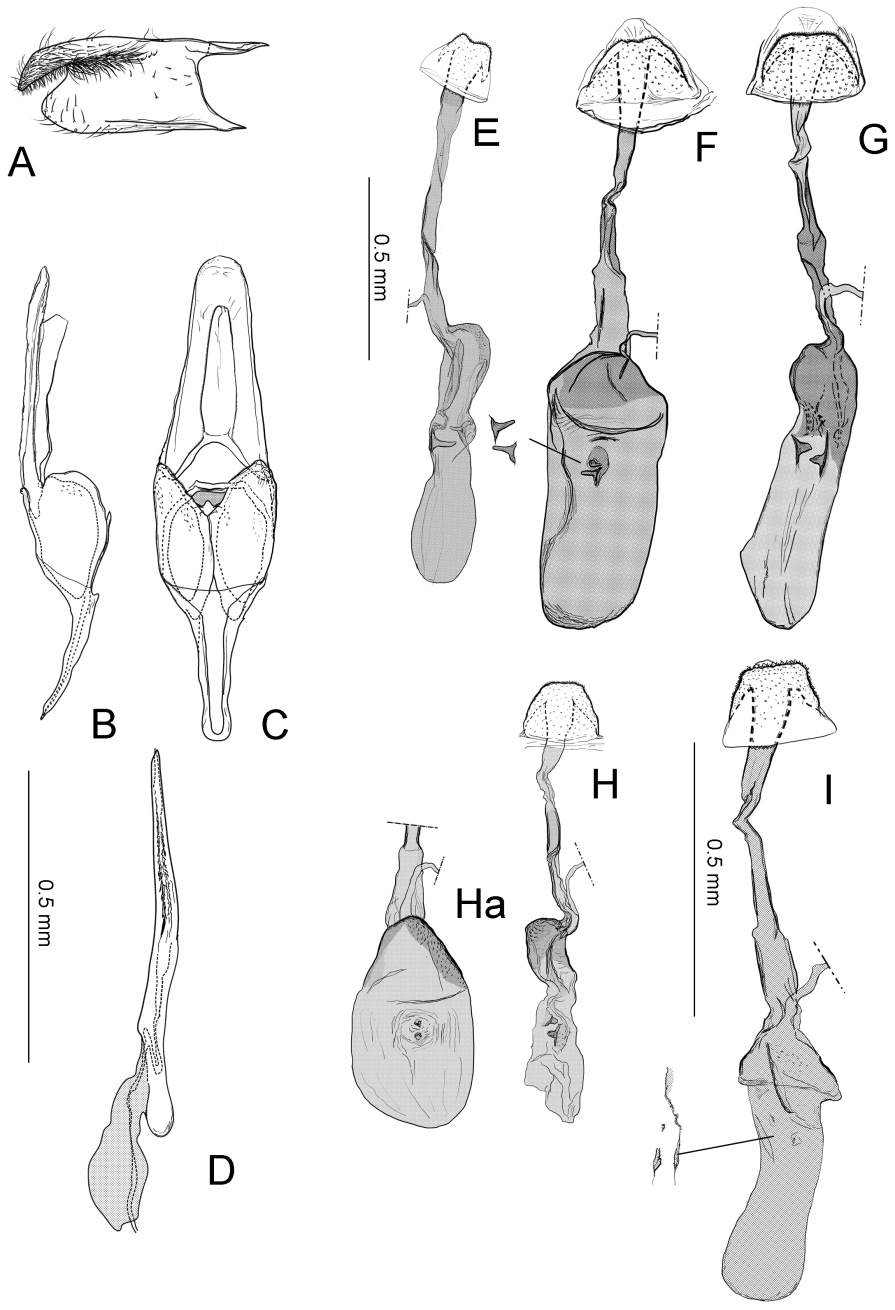
Genitalia of *Philodoria*. **E–I** Female. **A–D** *P. kolea* holotype male Hawaii SK851 **E** *P. succedanea* paralectotype Maui SK714 **F** *P. succedanea* leaf dropper Hawaii SK624 **G** *P. auromagnifica* non leaf dropper Hawaii SK623 **H** *P. kauaulaensis* holotype SK690 **I** *P. kolea* paratype Hawaii SK634. **A** Valva **B** Genital capsule lateral view **C** Genital capsule ventral view **D** Phallus lateral view

##### Distribution.

Kauai, Oahu and Lanai: new record, Maui (Walsingham 1907), Molokai and Hawaii (Big Island) (Zimmerman 1978).

##### Host plants.

Primulaceae: *Myrsine sandwicensis* A. DC., *M. lessertiana* A. DC. (Johns et al. 2016), *Myrsine* sp. (Zimmerman 1978). *Myrsine linearifolia* Hosaka and *M. knudsenii* (Rock) Hosaka are new host records (see Remarks).

##### Biology.

**Figure 8.**
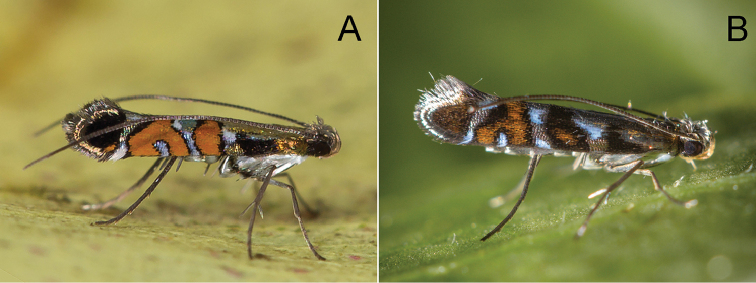
Resting posture of adult *Philodoria*. **A** *P. succedanea* Waikamoi Maui **B** *P. auromagnifica* Molokai CJ241.

**Figure 9.**
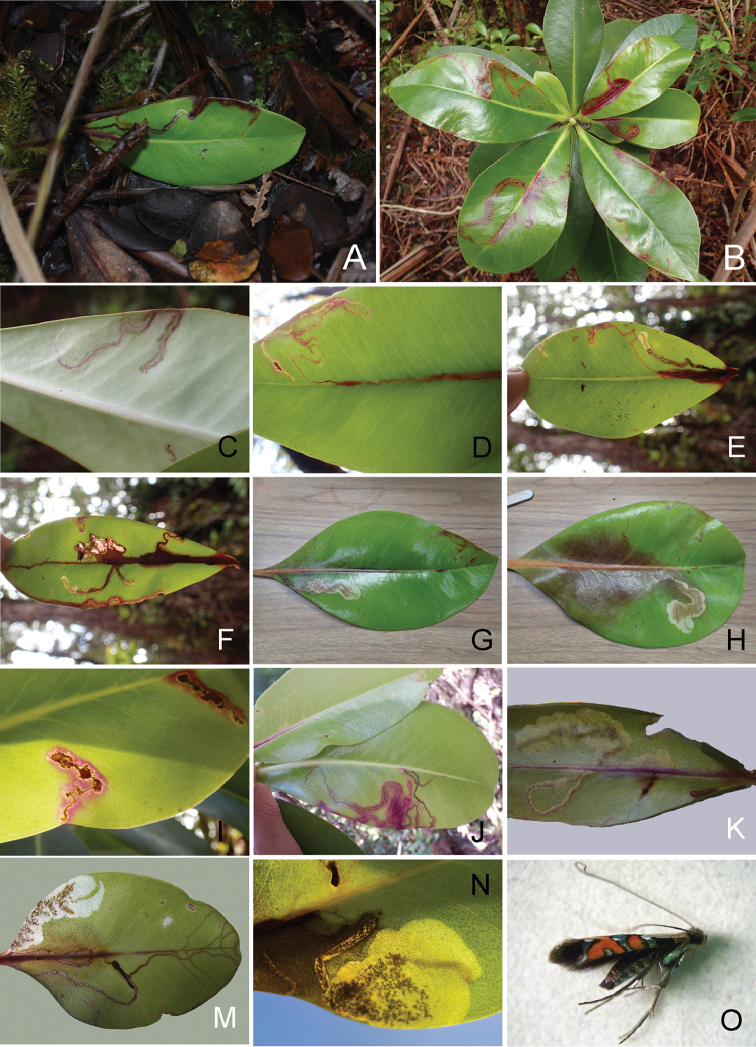
Biology of *Philodoria succedanea* with its hostplant, *Myrsine lessertiana*. **A–J** Hawai’i Volcanoes National Park, Hawaii (Big Island) **K–N** Maui **A** Fallen leaf and leaf mine with larva **B** Hostplants and leaf mines **C, I, J** Young mine **D** Leaf vein mine **E–G** Mine by late instar larva **H, K, M** Mature larva and mine **N** Mature larva **O** Adult, CJ-145, lateral view.

(Figs 8A, 9, 14A). The larvae mine the adaxial side of leaves of *Myrsine* species, forming a long linear mine (Fig. 9B, G, H). The mine is at first tornus-shaped (Fig. 9C, D, I, J) and the larva broaches the mid vein towards the petiole of the leaf, forming a straight mine; the vein mine and surrounding pattern are red in coloration (Fig. 9F, H) and later instars leave the mid vein usually near the base of the leaf, gradually expanding as they feed and grow forming a full-depth mine (Fig. 9E, F). There were usually one to two mines per leaf (Fig. 9B, G, M). The pupal cocoon is situated outside of the mine, usually on the leaf surface, and also on the woody tissue of the host plant with leaf mines and larvae. At Hawai’i Volcanoes National Park, larvae were collected from leaves that had fallen to the ground and reared to adulthood (Fig. 9A). The adult has been observed during the day (Maui and Hawaii Island), resting on the upper leaf surface of the host plant (Fig. 8A).

##### DNA barcoding.

BIN BOLD:ADF5435. The two specimens sequenced for COI, one from Maui and one from Hawaii, have identical DNA barcode sequences. The p-distance to the nearest neighbor, *P. kauaulaensis*, is 6.63%.

##### Remarks.

We identified two adult moths (Coll ID CJ-144 / GenBank accession no. ID KT982414 and CJ-145) as *P. succedanea*, based on the presence of a basal black patch on forewing, from which whole bodies were sacrificed for molecular analysis (Johns et al. 2016; Figs 6O, 12). Zimmerman (1978) did not recognize Walsingham’s (1907) Kauai record of this species because Walsingham had only one specimen at hand, which was in poor condition (specimen data: 1 ♂, Mts [which Mts not further specified], 3–4000 ft., Kauai, vi. 1894 Perkins.27297| PARATYPE 17/17 (?)|‘NOT succedanea Det. by E. C. Zimmerman|NHMUK010862812). We could not find the specimen from Kauai. However, we found *Myrsine knudsenii* (Endangered, IUCN) leaves with mines with active larvae from Kokee, Kauai Is. (CJ-433, 442), which were similar in appearance to mines of *P. succedanea* on *M. lessertiana*. Judging from these data, we consider the larval mines on *M. knudsenii* were made by *P. succedanea*. We also collected active *Philodoria* leaf mines from *M. linearifolia* (Endangered, IUCN) at the same location as *M. knudsenii*, but were unable to rear adult moths. It is thus possible that *P. succedanea* also mines *M. linearifolia*, but this needs to be further examined.

#### Philodoria kauaulaensis

Taxon classificationAnimaliaLepidopteraGracillariidae

Kobayashi, Johns & Kawahara sp. n.

http://zoobank.org/391CBA73-B2B0-462C-8608-0521E5B2572E

**Figure 10.**
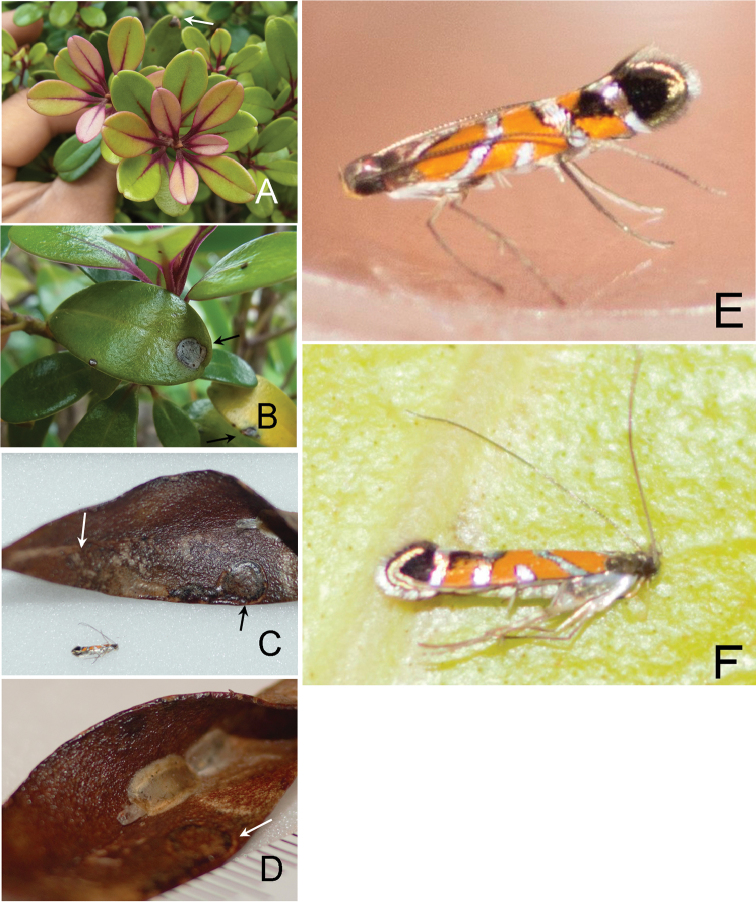
Biology of *Philodoria kauaulaensis* with its hostplant, *Myrsine lessertiana*, Haelaau, Maui, CJ-064. **A, B** Hostplant leaves and mines **C** Spiral mines, cocoon and adult moth **D** Cocoon with pupal exuvia **E** Resting posture of adult **F** Dead adult. Arrow showing leaf mine.

Figs 5I, J , 7H , 10 , 14B

##### Type locality.

Kauaula (Maui).

##### Type material.

Holotype ♀, Kauaula, Maui, 18.viii.2014 (stored in 99% ethanol), C.A. Johns leg., host: *Myrsine lanaiensis*, 31.vii.2014, CJ-381, SK690 in BPBM. The holotype is incomplete but we consider it distinctive enough to be worth describing. What remains of the holotype was mounted by placing three wings without mountant under a coverslip: two forewings (3/4 of right wing and half of left wing), and the apical portion of one hindwing (Fig. 5I). The head, antenna, thorax, and legs were sacrificed for molecular analysis.

##### Additional material.

2 unsexed (CJ-064, CJ-072), entirely sacrificed for molecular analysis and belonging to BIN BOLD:ADI5327 (See Remarks): 1 unsexed, Haelaau, Maui, 26.iv.2013 (stored), C.A. Johns leg., host: *M. lessertiana*, 8.iv.2013, CJ-064, KT982404; 1 unsexed, Haelaau, Maui, 29.iv.2013 (stored), C.A. Johns leg., host: *M. sandwicensis*, 8.iv.2013, CJ-072, KT982407.

##### Diagnosis.

The forewing pattern of this species is similar to that of *P. succedanea*, but differs from the latter by having broad orange transverse fasciae (Fig. 10E, F) and a white and bronze band near the apical portion of wing, in the middle interrupted by a blue patch (Fig. 5I, J).

##### Description.

**Adult** (Fig. 5I, J). Forewing length 2.4 mm, basal part of holotype forewing missing. Descriptions of the basal forewing and part of the body were based on photographs of adult moths (CJ-064, 072). Head and frons fuscous; maxillary palpus unknown; labial palpus ochreous. Antennae dark fuscous. Thorax unknown. Forewing shiny, metallic bronze with three large bright orange transverse fascia: an oblique one from costal fold to dorsal 1/4; a second at the middle of wing, narrowing greatly in the dorsum, containing a white costal spot; a third at 3/4 in the middle, interrupted by a blue patch; all fascia bordered with black scales: one white color band at middle of the first bronze color band, others on both extremities of third and fourth bands; a fuscous patch extending toward termen and apex with a black apical spot; cilia shiny, dark bronze grey. Hindwing dark tawny fuscous. Abdomen fuscous above, whitish beneath.

**Male genitalia.** Unknown.

**Female genitalia.** (Fig. 7H) (n = 1). Similar to *P. succedanea* and *P. auromagnifica*, but different in having rather smaller and rounded spines on the signa.

##### Distribution.

Maui.

##### Host plants.

Primulaceae: *Myrsine lanaiensis* Hillebr., *M. lessertiana* A. DC., and *M. sandwicensis* A. DC.

##### Biology.

(Figs 10, 14B). The mine is initially spiral-shaped (Fig. 10B, C) and gradually expands as the larva feeds and the mine later gets the form of a blotch (Fig. 14B). The pupal cocoon is situated outside the mine, usually on leaf surface (Fig. 10D).

##### DNA barcoding.

BIN BOLD:ADI5327. The two specimens sequenced for COI are from Maui and have a 0.17 p-distance between them, the p-distance to the nearest neighbor, *P. auromagnifica*, is 5.58%.

##### Etymology.

The specific epithet is derived from the type locality, Kaua`ula (pronounced ‘cow-wa-u-la’) Valley, an important site for Hawaiian endemic plants and culturally and spiritually for Native Hawaiians.

##### Remarks.

Johns et al. (2016) collected larvae from *Myrsine lessertiana* and *M. sandwicensis* in West Maui and identified the reared adult moths as *P. auromagnifica* (Coll. ID CJ-064 / GenBank accession no. KT982404 and CJ-072 / KT982407). Comparison of adult morphology and larval behavior with other species shows that these moths belong to *P. kauaulaensis* (Figs 10, 14). Unfortunately these specimens were sacrificed for molecular analysis, so that they cannot be added to the type series.

#### Philodoria auromagnifica

Taxon classificationAnimaliaLepidopteraGracillariidae

Walsingham, 1907

**Figure 11.**
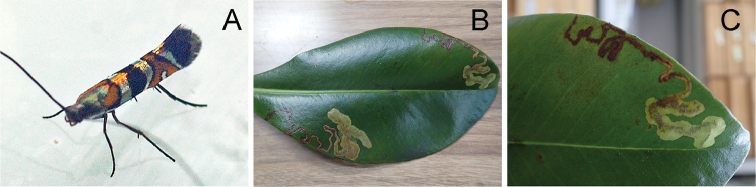
Biology of *Philodoria auromagnifica* with its hostplant. **A** Resting posture of adult male, host: *Myrsine wawraea* Kauai CJ-148 **B, C** Later mine, host: *M. lessertiana* Hawai’i Volcanoes National Park, Hawaii.

Figs 2E, F , 5E–H , 6E–H , 7G , 8B , 11 , 14C

Philodoria auromagnifica Walsingham, 1907: 718, pl. 25, fig. 20; Swezey 1913b: 223.
Philodoria (Philodoria) auromagnifica Walsingham, 1907: Zimmerman 1978: 695, figs 461, 468, 474.

##### Type locality.

mountains, 2000 ft near Honolulu (Oahu).

##### Type material.

Holotype ♂, Mts. 2000 ft near Honolulu, Oahu, 25.x.1892, Perkins. 25857|BM slide no. 472|Walsingham Collection. 1910–427.|NHMUK010305330| in NHMUK. This species was described based on a single specimen from Oahu. The ‘type’ specimen, designated by Walsingham is here thus the holotype following article 73.1.2 (ICZN 1999).

##### Additional material.

22 (8♂ 11♀ 3 unsexed)

Kauai Is: 1♂, Mt. Kahili, 18.vi.2013 (stored), N. Tangalin leg., Nat Collection, host: *M. wawraea*, CJ-148, SK689♂ in BPBM; 1♀, 4000 ft, Kokee State Park, Kahuamaa Flat, 21.viii.1973|K. & E. Sattler, BM1973-498|BMNH(E)1621087|*Philodoria* sp. 5 (Kauai) Sattler coll. Colour slide 67, D.C. Lees Sep 2016 in NHMUK; 1♂, same data labels as last specimen but 28.viii.1973|67|BMNH(E)1621087; 2 unsexed, Kauai, 3600’, Kokee State Park Kaumuohua Ridge (Milolii Ridge Rd) 1.vii.1982|K. & E. Sattler, BM1982-342| BMNH(E)1621081; same data, but BMNH(E)1621088; 1♂, Kauai, 3800’, Kokee State Park Kumuwela Ridge Waininiua Trail 24,vi.1982|K. & E. Sattler, BM1982-342|BMNH(E)1621091.

Oahu Is: 1♂, Kahana, 1.i.1928, O.H. Swezey Collector, “*Suttonia*“(= *Myrsine*), Z-XII-20-62-5♂, BPBM no. 34142 in BPBM; 2♀, Olympus, Coll. O.H.S, ex *Myrsine*, 33, J.F.G.C. #3801♀ in USNM. 1♀, Wailupe, 11.i.1925, O.H. Swezey Collector, “*Suttonia*“(= *Myrsine*), Z-XII-20-62-6♀, 34143 in BPBM.

Molokai Is, in BPBM: 1 unsexed, Kawela, 3700ft, 23.xii.1925, O.H. Swezey Collector, “*Suttonia*”(= *Myrsine*), 34144;1♂, Kamakou Boardwalk, 24.i.2014 (stored), C.A. Johns leg., host: *M. lessertiana*, 18.xii.2013, CJ-241, SK768♂ in BPBM.

Hawaii Is., Hawai’i Volcanoes National Park, host: *M. lessertiana* in BPBM: 2♀, A. Kawakita leg., “Non-leaf-dropper”, 25.iv.2016 (larva), SK622♀, SK623♀; 3♂ 4♀, 17–24.v.2016 em., A.Y. Kawahara leg., 27&29.iv.2016 (Cocoon & larva), SKH-10, SK802♀, SKH-13, SK805♂, AYK0002, SK806♂, HILO053, SK800♂, HILO054, SK811♀, HILO059, SK810♀; 1♀, Lava tube, 15.v.2016 em., C.L.-Vaamonde & C. Doorenweerd leg., 22.iv.2016, HILO020/AYK0001, SK809♀.

##### Diagnosis.

This species is very similar to *P. succedanea*, but recognizable by the dark brownish orange patches and brownish orange basal patch in the forewing; a fuscous patch with dark orangish scales in the apical portion (Table 4; Figs 2E, F, 5E–H); in the male genitalia by the rather long valva narrowing in the middle, vinculum large, inflexed on the ventral side, broad and straight saccus (Fig. 6E–G); in the female genitalia by signa with rather blunt spines (Fig. 7G). See also diagnosis of *P. succedanea*.

##### Redescription.

**Adult** (Fig. 2E, F). Wingspan 8 mm in holotype, 7–9 mm in other specimens; forewing length 3.5 mm in holotype, 3.2–3.9 mm in others. Head and frons dark steely fuscous; maxillary palpus reduced; labial palpus ochreous to brown. Antenna dark fuscous. Thorax: dark brownish orange, becoming fuscous posteriorly. Forewing shiny, metallic bronze with dark brownish orange patches: a large one at base bordered with black ground color (Figs 2E, F, 5E, F, H), sometimes missing orange color (Fig. 8B); an oblique transverse fascia before the middle of wing, bordered with black ground color, sometimes missing orange color (Fig. 11A); a large transverse patch after the middle to costal 3/4, narrowing greatly in the dorsum, extending to dorsal 2/3, containing a white costal spot; one white color band on the middle of the first bronze color band, others on both extremities of second and third bands; a fuscous patch mixed with dark brownish orange scales extending toward the termen and apex with a black apical spot; cilia shiny, dark bronze grey. Hindwing dark tawny fuscous. Abdomen and legs fuscous above, white beneath.

**Male genitalia** (Fig. 6E–L) (n = 5). Capsule 940–980 µm. Tegumen 540–580 µm long. Similar to *P. succedanea* except tegumen 1.2× length of valva; valva 460–480 µm long, broad and slightly narrowing in the middle (Fig. 6E, I); vinculum large, inflexed on the ventral side (Fig. 6G, K); saccus 300 µm long, broad and straight (Fig. 6F).

**Female genitalia** (Fig. 7G) (n = 7). Similar to *P. succedanea*, but different in having rather slender tapering antrum and rather blunt spines on the signa.

##### Distribution.

Kauai: new record, Oahu (Walsingham 1907), Molokai (Swezey and Bryan 1929), and Hawaii (Big Island) (Zimmerman 1978).

##### Host plants.

Primulaceae: *Myrsine* sp. (Swezey 1913a), *M. lessertiana* A. DC. and *M. sandwicensis* A. DC. (Johns et al 2016), *M. wawraea* (Mez) Hosaka: new record.

##### Biology.

(Figs 8B, 11, 14C). The larvae mine the adaxial side of leaves of *Myrsine* species, forming a long serpentine mine (Fig. 11B) and gradually expanding as they feed (Figs 11C, 14C2, C3). Old mines are ocherous to brown in coloration (Fig. 14C1). There were usually one to two mines per leaf (Fig. 11B). The pupal cocoon is prepared outside the mine, on either surface of the leaf, and one was found on the bark of the host.

##### DNA barcoding.

BIN BOLD:ADD6965. The two specimens sequenced for COI are from Hawaii and diverge by 0.31%, whereas the p-distance to the nearest neighbor, *P. kauaulaensis*, is 5.58%.

##### Parasitoids.

*Euderus metallicus* (Ashmead, 1901), Eulophidae (Zimmerman 1978).

##### Remarks.

We collected *Philodoria* leaf mines from *Myrsine* plants on Kauai Island (See also remarks for *P. succedanea*), only one male adult identified as *P. auromagnifica* emerged from a larva that fed on *M. wawraea* (Fig. 14C3). The Kauai specimens have a black second transverse fascia (Fig. 11A), but male genital variation that we observed appears to be intraspecific (Fig. 6I–L). Some specimens have a oblong valva which narrows in the middle (Fig. 6I), while others have a long tegumen about same length of valva, and slender vinculum and saccus in ventral view (Fig. 6J, K). We notice some wing pattern variation between islands, particularly in the extent of the orange forewing markings, and detailed DNA barcoding in future may prove revealing as regard the integrity of this species as we recognize it here. Two barcoded specimens collected from Hawaii (Big) Island (RMNH.5013750, CLV6240) belong to the same BIN (BOLD:ADD6965).

#### Philodoria kolea

Taxon classificationAnimaliaLepidopteraGracillariidae

Kobayashi, Johns & Kawahara sp. n.

http://zoobank.org/36268FAD-7EAE-4761-8EC4-87E19E7BF50E

**Figure 12.**
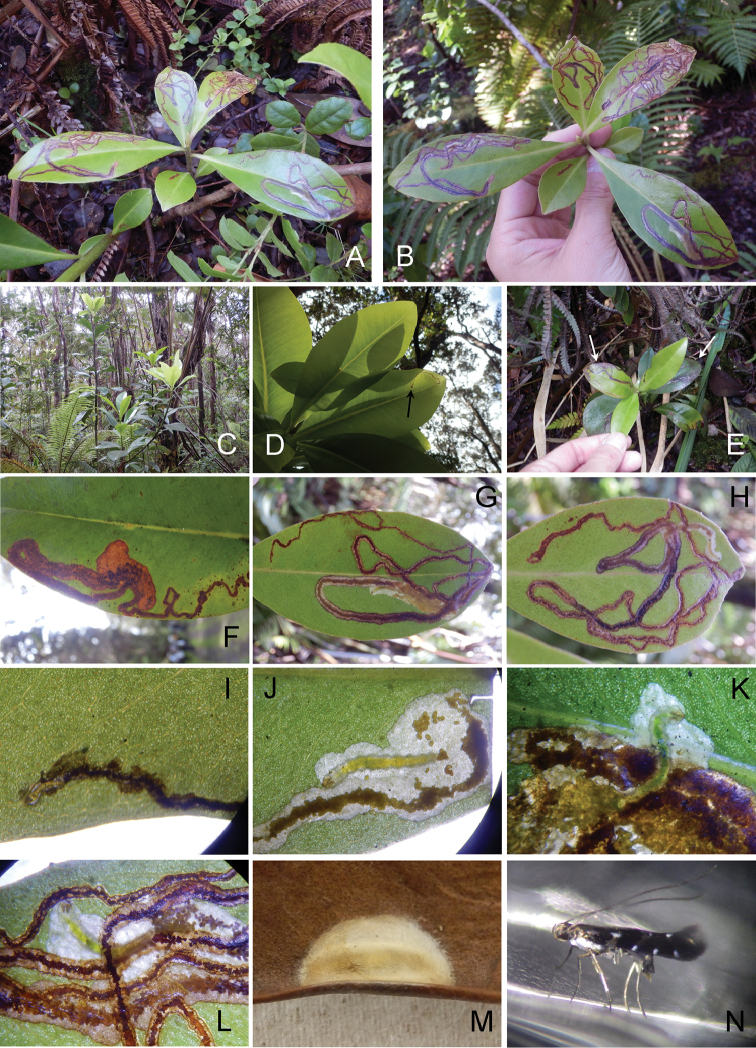
Biology of *Philodoria kolea* with its hostplant, *Myrsine lessertiana*. **A–M** Hawai’i Volcanoes National Park **A–B, E** Hostplant leaves and mines **C** Habitat and hostplants **D** Leaves and young mine **F–H** Later mines **I** Young larva **J–L** Mine by later instar larva **M** Cocoon **N** Resting posture of adult, paratype female lateral view.

**Figure 13.**
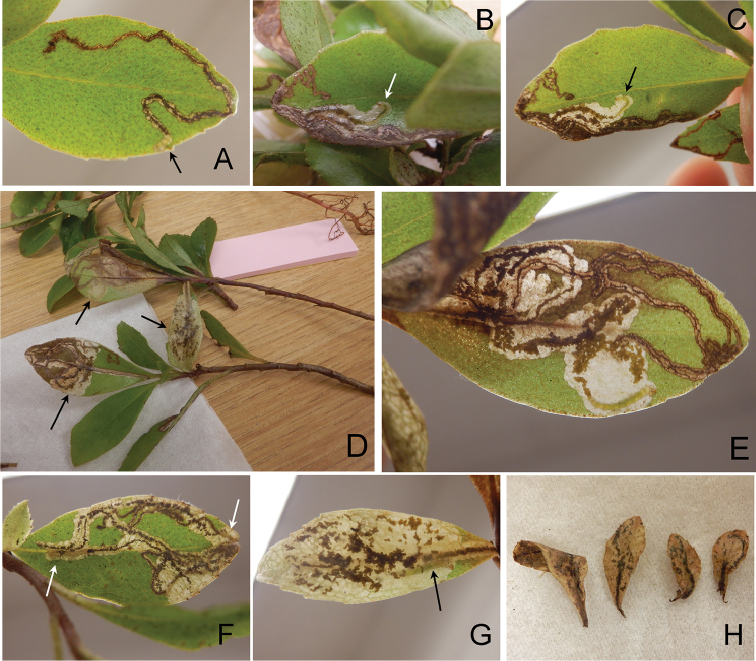
Biology of *Philodoria kolea* and its host, on seedlings of *Myrsine lessertiana*. **A** Young mine **B–C, E–F** Mine by later instar larva **G** Mature mine and larva **H** Full mature mine. Arrows showing larvae.

**Figure 14.**
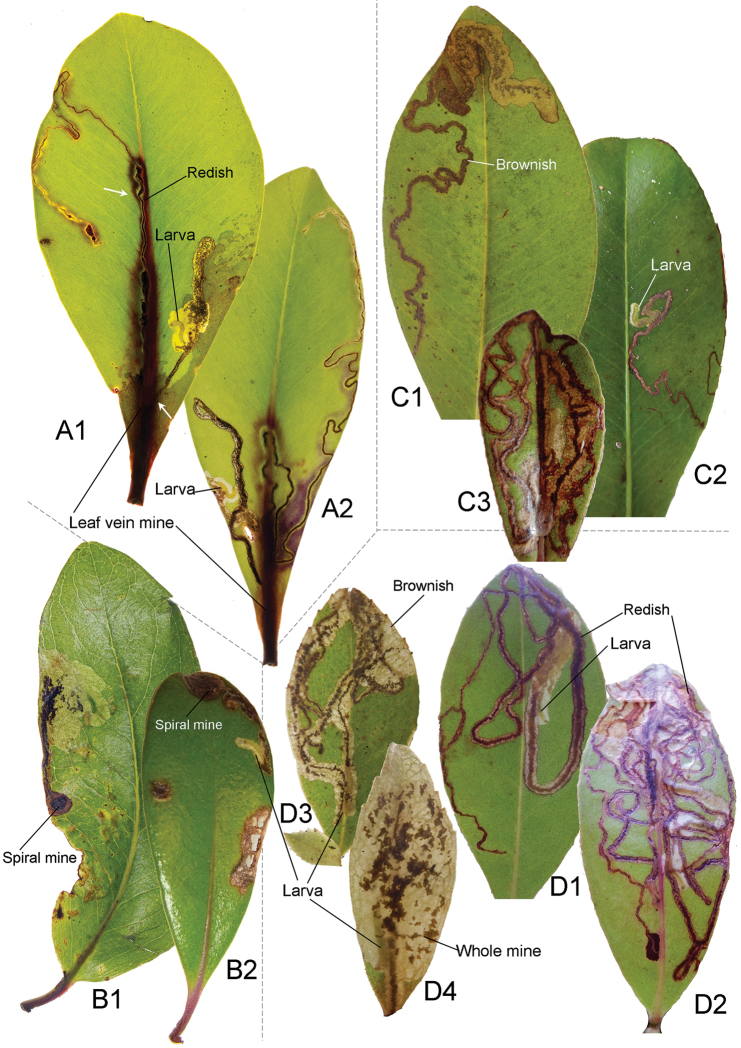
Mine forms and characters of *Philodoria* species and their *Myrsine* host plants. A: long linear form via mid vein ; B: Spiral to blotch form; C, D: Serpentine form. **A** *P. succedanea* **B** *P. kauaulaensis* **C** *P. auromagnifica* **D** *P. kolea* **A, C1–2, D** *M. lessertiana* **D1, 2** same collection of SKH-05-1 **B1** *M. lanaiensis*, same collection of CJ-381 **B2** *M. sandwicensis*, same collection of CJ-072 **C3** *M. wawraea*, CJ-148. **A** Molokai **B** Maui **C1–2, D** Hawaii **C3** Kauai.

Figs 3 , 5K, L , 7A–D, I , 12 , 13 , 14D

##### Type locality.

Hawai’i Volcanoes National Park (Big Island).

##### Type material.

Holotype ♂, Hawai’i Volcanoes National Park, Hawaii (Big Island), 25.iv.2016, A. Kawakita leg., host: *Myrsine lessertiana* (understory shrub), GenBank accession no. MF804825, IO-322, SK851 in BPBM. The type series was mounted from emerged adult moths.

Paratypes, in BPBM: 1♀, Kaumana Trail, Hilo, Hawaii (Big Island), 28.iv.2016, em., C.L.-Vaamonde & C. Doorenweerd leg., host: *Myrsine* sp., 20.iv.2016 (Cocoon), HILO016, SK634♀. 1♀, Thurston lava tube (Nahuku), Hawai’i Volcanoes National Park, Hawaii Is., 13.v.2016, em., S. Kobayashi leg., host: *Myrsine lessertiana*, 25.iv.2016 (larva), SKH-05-1, SK632♀; 1♀, same locality and data as holotype, IO-323, SK852; 2♀, same locality as holotype, 2&24.v.2016, em., C.L.-Vaamonde & C. Doorenweerd leg., host: *Myrsine lessertiana*, 22.iv.2016 (larva), HILO020/SKH-15, SK630♀, 631♀.

##### Diagnosis.

Among *Philodoria* species having similar fuscous forewing coloration (i.e., *P. wilkesiella* Swezey, *P. pipturiana* Swezey, *P. epibathra* (Walsingham), and *P. nigrella* (Walsingham) (See Zimmerman 1978)), the new species is recognizable by the white and bronze color bands on the forewing (Fig. 3). The forewing pattern and the genitalia are similar to those of other *Myrsine* mining species, *P. succedanea* and *P. auromagnifica*, but *P. kolea* completely lacks the orange markings (Figs 2, 3).

##### Description.

**Adult** (Figs 3, 5K, L, 12N). Wingspan 6.7 mm in holotype, 6.6, 8.5 mm in paratypes; forewing length 3.0, 3.1 mm in holotype, 2.7–4.0 mm in paratypes. Head leaden grey; frons whitish grey; maxillary palpus reduced; labial palpus greyish ochreous, terminal joint with fuscous band at middle and at apex. Antenna greyish fuscous. Thorax leaden grey. Forewing base leaden grey, externally suffused with brownish fuscous patches: a triangular basal patch along the costal fold; an oblique transverse fascia before the middle of wing, bordered with black scales; a large transverse patch after the middle to costal 3/4, narrowing in the dorsum, extending to dorsal 2/3, containing small white costal spot; leaden grey small median line at base with dorsal narrow patch from base to near middle; one white color band at the middle of the first bronze color band, others on both extremities of second and third bands; a leaden grey patch extending toward the termen and apex with small shiny black apical spots; cilia leaden grey with a black fringe basal line; tonal cilia with a shiny white fringe basal line. Hindwing and cilia leaden grey. Abdomen greyish fuscous above, banded with white beneath. Legs pale greyish fuscous, spurs white.

**Male genitalia** (Fig. 7A–D) (n = 1). Capsule 830 µm. Tegumen 600 µm long. Similar to *P. auromagnifica*, except tegumen 1.5× length of valva (Fig. 7A, C); valva 390 µm long, broad and having rather shorter and pointed dorsal process (Fig. 7A); saccus 250 µm long. Phallus 640 µm long.

**Female genitalia** (Fig. 7I) (n = 5). Similar to *P. succedanea* and *P. auromagnifica*, but different in having two very small and narrow signa with minute spines.

##### Distribution.

Hawaii (Big Island).

##### Host plants.

Primulaceae: *Myrsine lessertiana* A. DC.

##### Biology.

(Figs 12, 13, 14D). Larvae mine the adaxial side of leaves of *M. lessertiana*, forming a slender serpentine mine (Fig. 12A, B), and gradually expanding as they feed and grow forming a full-depth mine (Fig. 12G, L). Larvae consumed small amounts of leaf tissue (under 2 cm in leaf length) when feeding on seedlings (Fig. 13G, H). The young larva is about 1.5 mm long (Fig. 12I) and later instar larvae are 4–8 mm long (Fig. 12J, K). Larvae were collected from fresh leaves of seedlings. There was usually only one mine per leaf (Fig. 12A, B, G, H). The pupal cocoon is prepared outside the mine, on either surface of the leaf; the cocoon is greyish white to ochreous and near ellipsoidal in shape (Fig. 12M); 4.0–5.0 mm in length, 1.0–3.0 mm in width.

**Figure 15.**
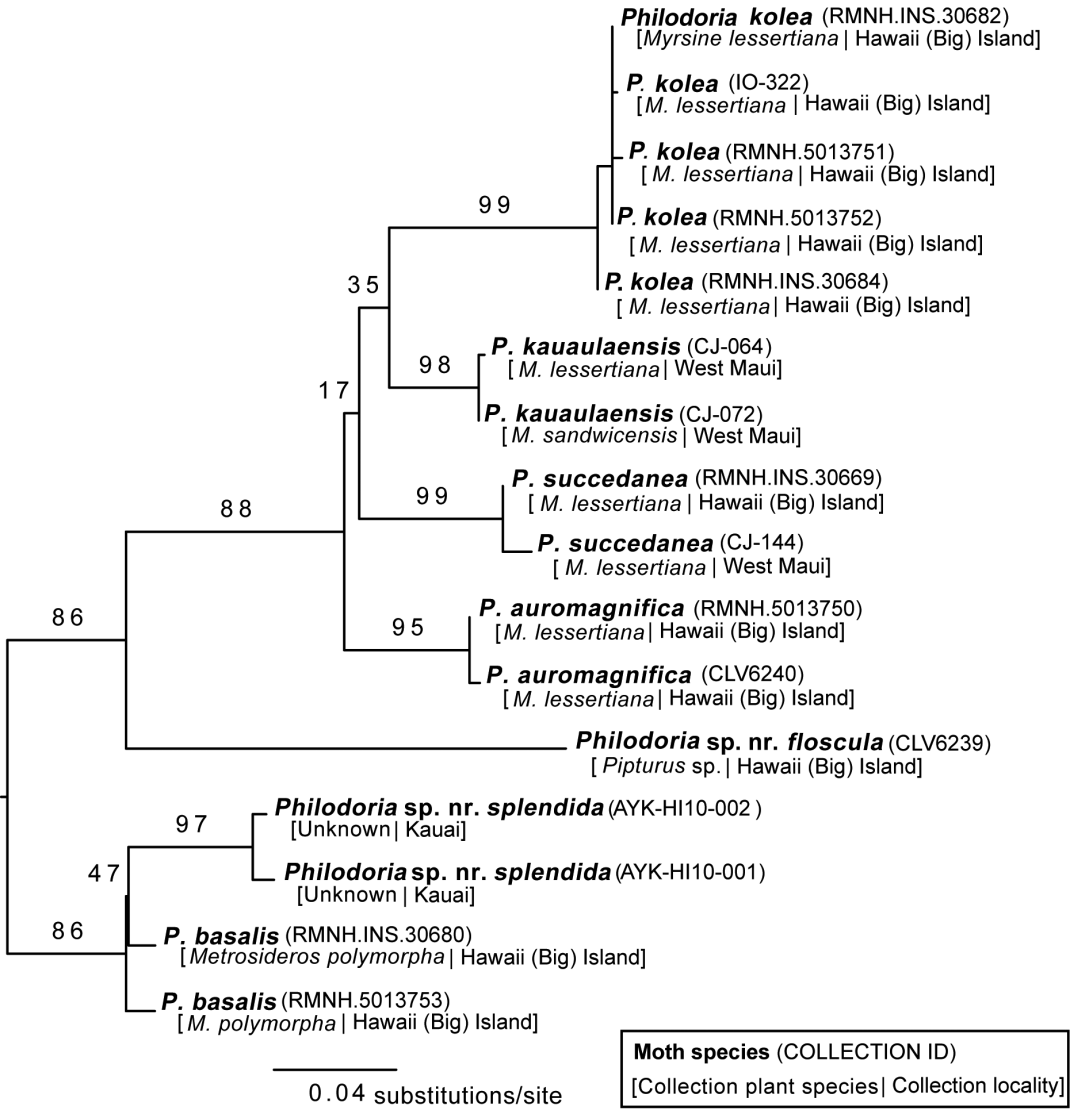
Maximum likelihood tree of *Philodoria* species based on the DNA barcoding region. Numbers on nodes indicate bootstrap support values. Collection ID, host plant, and collection locality, are also shown.

##### DNA barcoding.

BIN BOLD:ADF137. The five specimens sequenced for COI are from two localities on Hawaii and have maximum intraspecific p-distance of 0.17%. The p-distance to the nearest neighbor, *Philodoria kauaulaensis*, is 6.98%.

##### Etymology.

The specific epithet, *kolea*, is a noun in apposition taken from the Hawaiian name of the host plant, *Myrsine*.

### Molecular analysis

We obtained DNA barcode data for 16 individual specimens (http://dx.doi.org/10.5883/DS-PHDRIA). All species have their own unique cluster or Barcode Index Number (BIN) allowing their unequivocal identification (Table 2). The lowest interspecific distance (4.41%) was observed between Philodoria sp. nr. splendida and *P. basalis* (Table 3). Sequences of *Myrsine*-feeding *Philodoria* species, when compared pairwise, formed distinct clusters, with a maximum intraspecific divergence which varied between 0.17–0.88% and a NN distance which varied from 5.85–8.91% (Table 3). The minimum interspecific distance was smaller between *P. auromagnifica* and *P. kauaukaensis* (5.85%) than between *P. auromagnifica* and *P. succedanea* (6.71%) (Table 3). Identifying species using DNA barcodes appears to be useful for the *Myrsine*-feeding *Philodoria*. *Philodoria succedanea* belongs to BIN BOLD:ADF5435, *P. kauaulaensis* to BOLD:ADI5327, *P. auromagnifica* to BOLD:ADD6965, and *P. kolea* to BOLD:ADF7137.

## Discussion

Hawaiian *Philodoria* leaf mining moths were extensively studied in the early 1910s–1940s by Otto Herman Swezey. However, little taxonomic work has been conducted since, and our investigation is revealing that several undescribed cryptic species remain to be discovered, as found in other Hawaiian micromoths (e.g., *Bedellia*, Bedelliidae: [Zimmerman 1978]; *Hyposmocoma*: Cosmopterigidae [Kawahara and Rubinoff 2012; Rubinoff 2008]). *Philodoria* is critically in need of taxonomic work considering the endemic distribution of its species on the Hawaiian islands, and the close association of the genus with native endemic and endangered host plants. Some host plants and their associated *Philodoria* have already become locally extinct (Johns et al. 2014).

Swezey collected *Myrsine*-feeding *P. succedanea* and *P. auromagnifica* from numerous localities on Oahu in the early 1900s. *Myrsine lessertiana* plants remain relatively abundant on Oahu, but *Myrsine*-mining *Philodoria* have become exceedingly difficult to find there, especially in the southeast where intense urban development has taken place over the last century. During our Oahu surveys, we were unable to find leaf mines on *M. degeneri*, *M. fosbergii*, *M. juddii* (Critically Endangered, IUCN), *M. lanaiensis*, *M. pukooensis*, *M. punctata*, or *M. sandwicensis*, despite extensive searches for leaf mines on these host plants. It is not clear whether these absences are more due to environmental changes causing population reductions than to original host plant restriction among *Myrsine* species.

On Maui, *P. kauaulaensis* and *P. succedanea* were found in April–May 2013 at two sites separated only by 3.3 km, below the summit of Eke and on Haelaau Ridge, within the Pu’u Kukui Watershed Preserve (Fig. 2A; Johns et al 2016, fig. 1, Coll. ID CJ-064, CJ-072). In the present study, we observed *P. auromagnifica*, *P. kolea* and *P. succedanea* occurring in sympatry on April 2016 at the Hawai’i Volcanoes National Park, the island of Hawaii (Big Island) (Figs 2A, 6A–J, 8B, C, 9A–M).

We collected larvae of *P. auromagnifica* (Fig. 8B, C) on plants that were also used by *P. succedanea* (Fig. 6A). The latter species was still mining leaves from the same plants that had fallen to the ground. Larvae of *P. kolea* occurred on leaves that were intact on short (about 10–20 cm high) *Myrsine* plants at the same site (Figs 9A, B, 10D). The genetic similarity between these species could imply that perhaps competition and niche partitioning may have been the cause of speciation. Fine-scale niche partitioning has been documented in other gracillariids and their host plants, such as *Phyllocnistis* on *Persea* (Davis and Wagner 2011) and *Phyllocnistis* on *Salix* (Kobayashi et al. 2011). Our ongoing research efforts will examine the evolutionary history and colonization patterns of *Philodoria* on the Hawaiian archipelago.

In addition to providing morphological and molecular evidence to delimit species limits among the Hawaiian *Myrsine*-feeding *Philodoria*, we include a pictorial key to their leaf mines (Fig. 14). We include this information as leaf mining moths can be difficult to observe as larvae or adults to a non-specialist. Larvae of *P. succedanea* form red, long linear mines along the leaf vein (Fig. 14A), *P. kauaulaensis* produces at first spiral and later blotch mines (Fig. 14B), *P. auromagnifica* makes brown serpentine mines (Fig. 14C), and *P. kolea* creates complete serpentine mines fully occupying the adaxial side of leaf surface of *Myrsine* seedlings (Fig. 14D). We hope that local Hawaiian park rangers, naturalists, and educators can use this key as a means to identify these species, so that the collection of these much-needed data can persist.

It is likely that detailed molecular work among islands will reveal further cryptic species but native hostplants and habitats are under great threat.

## Supplementary Material

XML Treatment for Philodoria succedanea

XML Treatment for Philodoria kauaulaensis

XML Treatment for Philodoria auromagnifica

XML Treatment for Philodoria kolea
